# Enduring Effects of Early Life Stress on Firing Patterns of Hippocampal and Thalamocortical Neurons in Rats: Implications for Limbic Epilepsy

**DOI:** 10.1371/journal.pone.0066962

**Published:** 2013-06-18

**Authors:** Idrish Ali, Patrick O'Brien, Gaurav Kumar, Thomas Zheng, Nigel C. Jones, Didier Pinault, Chris French, Margaret J. Morris, Michael R. Salzberg, Terence J. O'Brien

**Affiliations:** 1 Department of Medicine, the Royal Melbourne Hospital, University of Melbourne, Victoria, Australia; 2 INSERM U1114, Physiopathologie et psychopathologie de la schizophrénie, Strasbourg, France; 3 Fédération de Médecine Translationnelle de Strasbourg, Université de Strasbourg, Strasbourg, France; 4 Department of Pharmacology, School of Medical Sciences, University of New South Wales, New South Wales, Australia; 5 Department of Psychiatry, St. Vincent's Hospital, University of Melbourne, Victoria, Australia; 6 Department of Neurology, the Royal Melbourne Hospital, Melbourne, Victoria, Australia; University Paris 6, France

## Abstract

Early life stress results in an enduring vulnerability to kindling-induced epileptogenesis in rats, but the underlying mechanisms are not well understood. Recent studies indicate the involvement of thalamocortical neuronal circuits in the progression of kindling epileptogenesis. Therefore, we sought to determine *in vivo* the effects of early life stress and amygdala kindling on the firing pattern of hippocampus as well as thalamic and cortical neurons. Eight week old male Wistar rats, previously exposed to maternal separation (MS) early life stress or early handling (EH), underwent amygdala kindling (or sham kindling). Once fully kindled, *in vivo* juxtacellular recordings in hippocampal, thalamic and cortical regions were performed under neuroleptic analgesia. In the thalamic reticular nucleus cells both kindling and MS independently lowered firing frequency and enhanced burst firing. Further, burst firing in the thalamic reticular nucleus was significantly increased in kindled MS rats compared to kindled EH rats (p<0.05). In addition, MS enhanced burst firing of hippocampal pyramidal neurons. Following a stimulation-induced seizure, somatosensory cortical neurons exhibited a more pronounced increase in burst firing in MS rats than in EH rats. These data demonstrate changes in firing patterns in thalamocortical and hippocampal regions resulting from both MS and amygdala kindling, which may reflect cellular changes underlying the enhanced vulnerability to kindling in rats that have been exposed to early life stress.

## Introduction

Mesial temporal lobe epilepsy (MTLE) is the most common form of focal epilepsy in adults, and is often drug resistant [1]. It is characterised by focal limbic seizures that commonly impair consciousness and may generalise to become convulsive in nature. The exact mechanisms underlying the spreading of seizures to become secondarily generalised are not known [2]. In animal models, early life stress has been shown to affect neuroplasticity, neuroendocrine and neurochemical functions as well as electrophysiological properties in structures that are relevant to MTLE, notably the hippocampus [3], [4]. These changes may be associated with the pathogenesis of MTLE [5], [6], [7], [8] and its associated psychiatric comorbidities, including anxiety related disorders [9]. Moreover, early life stress induced by maternal separation (MS) has been shown to lead to an increased vulnerability to amygdala kindling-induced epileptogenesis in rats [10], [11], [12], an important model of human MTLE [13], [14]. The effect of early life stress to result in an enhanced vulnerability to epileptogenesis has also been shown in other models of MTLE using different stress paradigms [15], [16], discussed in [4]. However, as yet there is little evidence on the underlying mechanisms for this.

Kumar et al [10] showed that early life stress induced by MS results in adult rats that show an acceleration in the progression of focal limbic seizures to secondary generalised convulsive seizures using the rat amygdala kindling model. There is evidence that thalamic structures play a critical role in the progression of kindled seizures in rats [17]. The GABAergic thalamic reticular nucleus (TRN), a key component of thalamocortical circuits, has been implicated by other work as being associated with the progression of limbic epileptogenesis [18]. Consistent with this, the administration of carbachol, a muscarinic receptor agonist, into lateral thalamic regions including the ventrobasal complex of thalamus and TRN was able to stimulate limbic and generalized convulsive seizures [19], [20].

We hypothesised that exposure to amygdala kindling would alter neuronal firing patterns in hippocampal and thalamocortical regions, and that exposure to MS would exacerbate these changes. Using in vivo juxtacellular recordings in rats, we found that interictal firing patterns of TRN neurons were altered by MS and kindling, while hippocampal pyramidal neurons were influenced only by MS. These alterations involve enhanced burst firing suggesting increased excitability of these structures. Consistent with this, during the initiation and progression of a stimulated seizure, the increase in the burst firing in somatosensory cortex was more pronounced in MS-exposed rats compared to early handled (EH) rats.

## Materials and Methods

### Ethics Statement

The study was approved by the Animal Ethics Committee of the Royal Melbourne Hospital, the University of Melbourne (Ethics number: 0911087) and conformed to National Health and Medical Research Council Guidelines for the ethical use of animals in scientific research. All efforts were made to minimize stress and the number of animals necessary to produce reliable data.

### Experimental animals

Wistar rats were bred and housed in plastic boxes in the Zoology animal facility, University of Melbourne during the period of early-life manipulations (see below). At five weeks male rats were transferred to Department of Medicine (RMH) University of Melbourne, Biological Research Facility for all other experiments. Animals were maintained in standard conditions of 12 hour light/dark cycle at 19–24°C with ad libitum access to food and water.

### Early life interventions

The early life stress protocol was based on the same methodology as our previous published work [10], [11], [12]. On the day of birth, postnatal day 0, pups were assigned to either MS or EH control group. Separations were done for 180 minutes for MS (approximately between 0800 to 1100 hr) and 15 minutes for EH (approximately between 0800 to 0815 hr) per day from postnatal day 2–14. During the separation period, the dam was moved to a different room while the pups were moved to a separate cage placed on a heating pad maintained at 30°C. At the end of the separation, the pups and the dams were returned to the original cage. Cages were cleaned and replaced on PN day 2, 7, 14 and 21. Pups were weaned on day 21, thereafter, left to normal housing conditions; 3–4 rats in each cage and cleaning of cages twice a week. We used rats from 15 litters with 8–12 pups in each litter. For 7 of the litters the MS pups were isolated from their siblings as well as their mothers during which time they were kept on tissue paper lined plastic boxes kept on a heating pad. Each breeding pair was used only once to avoid transfer of possible maternal stress induced effects to the next litter.

### Open field test

At 6 weeks of age, the open field test was performed to compare the anxiety-like behaviours between MS and EH rats, as previously described [21]. Briefly the rats were placed gently in the centre of an open arena of 1 metre diameter. Trials consisted of 5 minutes during which the rats were allowed to explore. The total distance travelled, and the frequency of entry and time spent in the central area of the field (diameter 66 cms) were quantified using Ethovision tracking software (Noldus Information Technology, Wageningen, The Netherlands).

### Bipolar electrode implantation and amygdala kindling

At 7 weeks of age electrode implantation surgery was performed [6]. Briefly, under isoflurane anesthesia, each rat was implanted with a bipolar electrode in the left basolateral amygdala, along with three epidural EEG electrodes and one anchoring screw, all held in place with dental acrylate. All the epidural electrodes for EEG recording were implanted on the left hemisphere, while the right hemisphere was left clear to be used for electrophysiological recordings. The dental acrylate was cleared over the bregma, to facilitate stereotaxic guidance during later experiments.

After one week recovery, the after discharge threshold was tested as previously described in [11]. Briefly, a current of increasing intensity was applied for 1 second through the bipolar electrode, using Accupulser Pulse generator/Stimulator, (A310, World Precision Instruments, Sarasota, FL), initially starting at 20 µA (60 Hz, 1 ms pulses) and increasing with 20 µA every 30 seconds, until an afterdischarge of at least 6 seconds was observed on EEG. Amygdala kindling started the next day: electrical stimulations were delivered at the after discharge threshold current twice daily, with an inter-stimulus interval of at least 4 hours. Seizures were graded according to the Racine's Classification [22], and the EEG recorded for each seizure. Preictal EEG recordings were also taken to characterise EEG parameters in freely moving rats. Kindling stimulation continued until rats were fully kindled (defined as 5 class V seizures) [22], after which the rats were used for electrophysiological experiments.

### Electrophysiology Experiments

#### Anesthesia and Surgery

Once a rat was fully kindled, it was used for electrophysiology experiments within two days. The rats were anesthetised using ketamine (100 mg/kg; i.p. Ketasol®; Richterpharma) and xylazine (10 mg/kg; i.p. Rompun®; Bayer). Rats underwent tracheotomy to facilitate artificial ventilation during the recordings (SAR-830, CWE, Ardmore, USA; in pressure mode (8–12 cm H_2_O, 60 breaths per minute)). Rats were placed in a stereotaxic frame and artificially ventilated. The body temperature was maintained between 36.2–37.2°C with a thermoregulated blanket (Fine Science Tools., Inc, Heidelberg, Germany) at all times during the recordings. The critical physiological parameters like heart rate (300–350 beats/minute) and arterial PO_2_ and PCO_2_ were monitored and maintained at all times. The recordings were conducted under neuroleptic analgesia, with the depth of anesthesia titrated to maintain predominantly desynchronized EEG. To facilitate administration of neuroleptic analgesia, penile vein catheterisation was performed. An initial dose of neuroleptic analgesia was administered: (0.25 ml in 10 minutes, of: d-tubocurarine chloride (0.2 mg; Sigma Aldrich), fentanyl (0.22 µg; Mayne Pharma), haloperidol (25 µg; Janssen)) i.v. through the penile vein catheter. Thereafter, it was maintained at a continuous i.v. infusion (0.5 ml/Hr) of the mixture: d-tubocurarine (0.4 mg), fentanyl (0.425 µg), haloperidol (50 µg) and glucose (25 mg; Sigma). Two craniotomies were performed followed by small incisions in the dura, through which electrodes were inserted targeting the three regions of interest: S1 somatosensory cortex, hippocampal CA1 and CA3 pyramidal neurons and TRN using the stereotaxic atlas [23], [24].

#### *In vivo* juxtacellular recordings

The firing patterns of S1 somatosensory cortex, hippocampal CA1 and CA3 pyramidal neurons and caudal region of the TRN neurons were recorded along with the EEG from frontal cortex and basolateral amygdala. The *in vivo* extracellular electrophysiology recordings were performed according to the methods described in [25]. Micropipettes were prepared from 1 mm thick glass capillaries (Harward Apparatus, Ltd., UK) by pulling, using a horizontal pipette puller (Sutter Instruments, CA; USA) and filled with a solution of 1.5% N-(2 aminoethyl) biotinamide hydrochloride (Neurobiotin, Vector Laboratories, Burlingame, CA; USA) in 1 M potassium acetate. Micropipettes (tip diameter ∼1 µm and resistance 20–30 mΩ) were lowered through the small incision in dura, using motorised stereotaxic microdrivers (StereoDrive, Neurostar, Germany). The data were processed (Cyberamp, Axon Instruments, Foster city, CA; USA) with band passes of 0.1–800 Hz for EEG and 0–6000 Hz for cellular activity. Data were then digitised at a sampling rate of 20 kHz per channel (Digidata 1440; Axon Instruments). The baseline cellular activity was recorded for 10 minutes after which a seizure was induced by stimulation using the bipolar electrode, followed by another 10 minutes recording. This allowed the study of firing patterns during interictal states as well as during a stimulated seizure. The last recorded pairs of neurons were labelled to confirm their location using the juxtacellular technique [25]. Briefly, the recorded cell was juxtacellularly filled with neurobiotin by applying positive currents (0.5–8 nA) through the micropipette tip (200 ms on/200 ms off for around 10 minutes). After the labelling, the micropipettes were slowly withdrawn, taking care not to injure the cell.

#### Histology

At the end of labelling the animals were killed with an overdose of pentobarbital (Lethobarb, Virbac; Australia) and were transcardially perfused with 200 ml of 0.1 M phosphate buffered saline (PBS) and fixed with 4% paraformaldehyde (PFA) solution in 500 ml PBS. The brain was extracted and was kept in PFA overnight for post fixing. Coronal sections of 100 µm thickness were cut using the Vibrotome (Vibrotome^TM^, St Louis, MO; USA) and serially collected in 0.1 M PBS. The sections were thoroughly washed with PBS and incubated overnight with a solution of 1∶100 avidin-biotin-peroxidase complex solution (Vectastain, ABC kit; Vector Laboratories, Burlingame, CA; USA) and 0.3% Triton X-100 at room temperature. The tracer was then revealed with nickel intensified 3,3 P-diaminobenzidine tetrahydrochloride activated by peroxidase (DAB; Vector Laboratories, Burlingame, CA; USA). The sections were then mounted on gelatin coated slides. These slides were then treated with 0.1% thionin for 30 seconds, washed with water for 1 minute, dehydrated and cover slipped. The location of the bipolar electrode and any labelled cells was verified microscopically referring to the stereotaxic atlas [23].

### Data Analysis

#### EEG analysis

Fast Fourier transform were computed using Clampfit software Version 10.2 (Axon Instruments, Foster City, CA; USA) for frontal cortical EEG. For rats under neuroleptic analgesia, total power of each EEG band was based on 4-s epochs with a resolution of 0.49 Hz and was applied for at least 1 minute of EEG. Total power of each EEG band in freely moving rats (recorded 1–2 days before the rats were used for electrophysiology experiments) was based on 1-s epochs with a resolution of 0.49 Hz and was applied for at least 30 seconds of EEG segments. The EEG bands were classified for 1–4 Hz (delta), 5–9 Hz (somatosensory rhythm), 10–16 Hz (spindle), 17–29 Hz (beta), 30–80 Hz (gamma) [26].

#### Neuronal firing frequency and Burst firing

Data analysis was done using the Clampfit software Version 10.2 (Axon Instruments, Foster City, CA; USA). During interictal states, a total of three one minute epochs were analysed and averaged for each cell. Any cells recorded from the somatosensory cortex or the hippocampus that had firing characteristics based on their firing frequency and action potential duration similar to that of an interneuron [27], [28] were excluded from the analysis. The action potentials recorded could fire as single action potentials or in burst mode. The inclusion criteria for a burst was a minimum of two action potentials, with an inter action potential interval of less than 6 ms duration. We determined the mean frequency of the neuronal firing and percentage of action potentials that fired in burst compared to the discrete events. We also calculated the characteristics of burst firing like mean number of action potentials in a burst, maximum number of action potentials in a burst and intraburst frequency. Similarly, mean neuronal firing frequency and % burst firing were analysed during the progression of a stimulated seizure.

#### Rhythmicity of firing

To assess whether the discharges of action potentials recorded were rhythmic, a method described previously [29] was used. This method involves construction of an autocorrelogram followed by making the spectrum (Lomb) of this autocorrelogram to detect the frequency as well as the statistical significance of the rhythmicity at observed frequency. The discharge patterns for which the peaks in the spectrum of autocorrelogram had a p<0.05 were considered to be rhythmic in nature. The automated software for analysing this data was generously provided by Dr. Kaneoke (Department of Integrative Physiology, National Institute of Physiological Sciences, Okazaki; Japan). The time stamps of action potential firing (start time) from 3–4 minutes of recording were obtained using Clampfit; using these data, the software was employed to determine whether each cell displayed rhythmic firing.

### Statistics

The parameters of anxiety-like behaviour were analysed using two-tailed Student's *t*-test. Neuronal firing patterns during progression of stimulated seizures were analysed using two-way ANOVA with repeated measures, with early life exposure and kindling being the two independent variables. The firing frequency, burst firing (including bursting characteristics) and EEG power of specific frequency bands were analysed for statistical differences between groups using two-way ANOVA. Post hoc planned comparison was used to determine the intergroup statistical differences. For binary variables (i.e. proportion of cells that had burst firing or rhythmic firing), Fisher's exact test was used to determine the statistical significance between the groups.

## Results

### Exposure to MS results in increased anxiety-like behaviour

**Figure 1:**
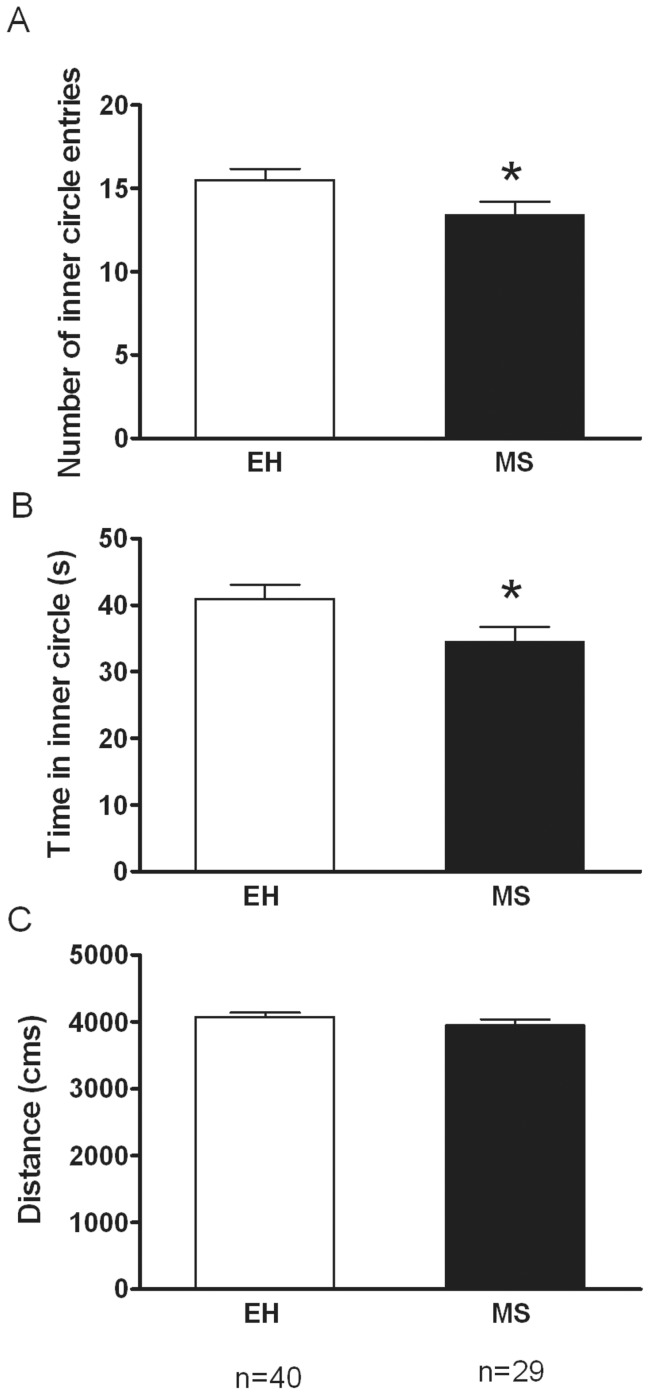
Effect of Maternal separation (MS) on anxiety-like behavior. Maternal separation (MS), increases anxiety-like behaviour on the open field test, compared to early handled (EH) controls. This was evidenced by significant reductions in (**A**) the number of inner circle entries (t test p = 0.04) and (**B**) the total time spent in inner circle (p = 0.04). No significant differences (p>0.05) were observed when comparing the total distance travelled (**C**). Data are expressed as Mean ± SEM.

Anxiety-like behaviour was tested at 6 weeks of age by using the Open Field Test. Compared to EH rats, MS rats showed a significantly increased anxiety-like behaviour as evidenced by a significantly lower number of inner circle entries (p = 0.046) as well as less total time spent in the inner circle (p = 0.037) (Figure 1). There was no significant difference in total distance travelled between MS and EH rats (p = 0.28).

### Kindling increases power of delta frequency activity EEG spectra

Fully kindled MS (median-23.0 and range 12 to 31 stimulations to fully kindled) and EH (median-25.5 and range 14 to 38 stimulations to fully kindled) rats along with sham kindled rats from both treatment were used for *in vivo* electrophysiology experiments.

**Figure 2:**
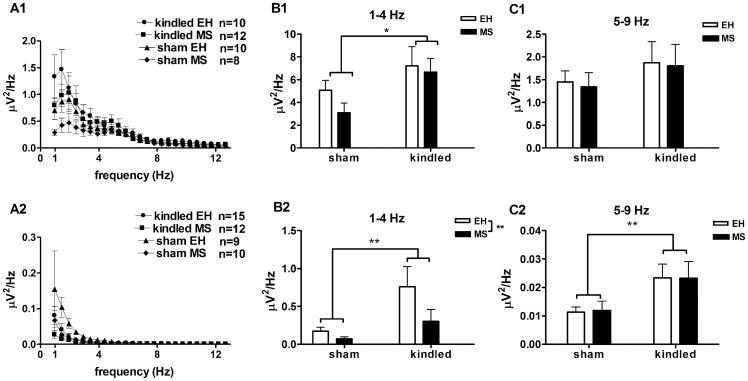
Effects of MS and Kindling on EEG spectral activity. EEG spectral activities were analysed from rats under neuroleptic analgesia (A1–C1) as well as freely moving rats (A2–C2). Total power in the low frequency delta 1–4 Hz band was significantly increased in kindled rats, compared to sham-kindled rats when measured in rats under neuroleptic analgesia (F_(3,34)_ = 11.9, p = 0.04, panel B1) and in freely moving rats (F_(3,47)_ = 15.4, p = 0.004, panel B2). Also, in freely moving rats, MS led to a significant decrease in the power of delta frequency activity (F_(3,47)_ = 7.26, p = 0.04, panel B2). In the 5–9 Hz frequency band, there were no significant effect of MS or kindling observed in rats under neuroleptic analgesia (C1), but in freely moving rats, there was a significant increase in power of 5–9 Hz frequency in kindled rats, compared to sham-kindled rats (C2). There was no significant effect of kindling or MS on power of higher frequencies (p>0.05; data not shown). Data are expressed as Mean ± SEM.

The spectral power of the frontal EEG was computed for the segments of the recordings from which the juxtacellular firing patterns were analysed (Figure 2A1). There was a significant increase in the power of 1–4 Hz (delta) frequency bands in the EEG of kindled rats (F_(3,34)_ = 11.9, p = 0.04), compared to sham-kindled rats (Figure 2B1). No other frequency bands were significantly affected by either MS or amygdala kindling (p>0.05). To check whether these results were not specific to the experimental conditions, a similar analysis was conducted on data obtained from freely moving rats (Figure 2A2). Similar to the rats under neuroleptic analgesia, there was a significant increase (F_(3,47)_ = 15.4, p = 0.004) in the power of 1–4 Hz (delta) frequency band in the EEG of kindled rats, compared to sham-kindled rats. Surprisingly, MS led to a significant decrease (F_(3,47)_ = 7.26, p = 0.04) in the power of delta band in the freely moving condition, compared to EH rats (Figure 2B2). In addition, there was a significant increase (F_(3,47)_ = 19.9, p = 0.001) in the power of 5–9 Hz in kindled rats, compared to sham-kindled rats (Figure 2C2). No other frequency bands were significantly affected by either amygdala kindling or MS (p>0.05).

### MS and kindling affect interictal neuronal firing patterns in the TRN and hippocampus, but not S1somatosensory cortex

The *in vivo* juxtacellular recordings were performed under neuroleptic analgesia from the caudal part of the TRN, pyramidal layers of the hippocampus including CA1 and CA3 neurons (data combined as there was no statistically significant difference in the firing patterns between these subregions) and S1 somatosensory cortical neurons (data from superficial (layer I–IV) and deeper layers (layer V–VI) were combined as their firing patterns were not statistically different).

#### Firing frequency

**Figure 3:**
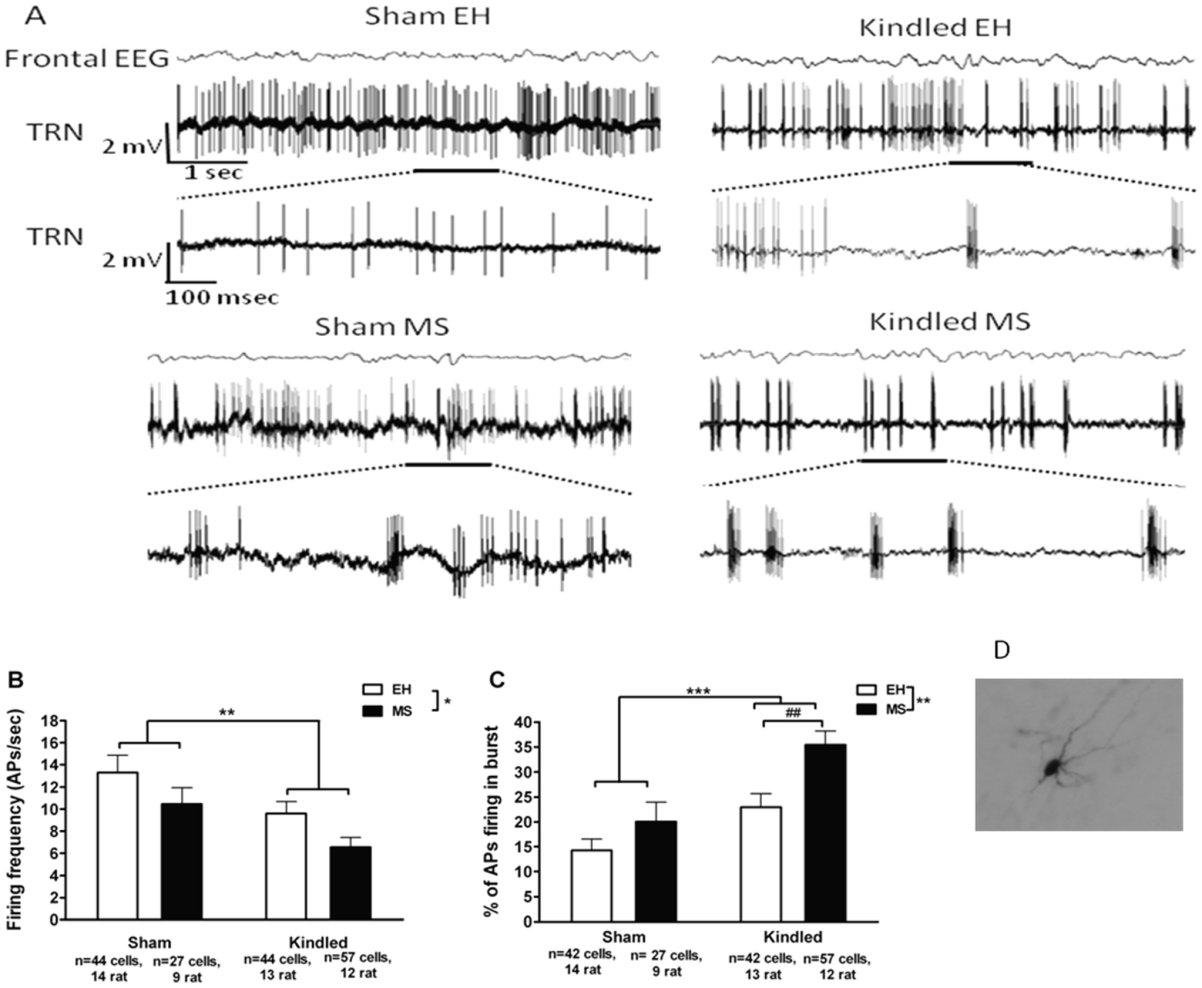
Effects of kindling and MS on interictal neuronal firing in thalamic reticular nucleus (TRN). Representative examples of recordings from each of the four groups are depicted in A with the underscored region of each upper trace expanded in the lower trace. **B** Both MS and kindling led to significant reductions in TRN neuronal firing frequency (F_(3,168)_ = 5.41, *p = 0.02 for MS and F_(3,168)_ = 8.93, **p = 0.003 for kindling), and these effects were accompanied by significant increases in the percentage of action potentials that fired in bursts (F_(3,165)_ = 9.24, **p = 0.002 for MS and F_(3,165)_ = 16.24, ***p<0.001 for kindling, panel C). Post hoc planned comparison showed that the percentage of action potentials firing in bursts were significantly more in MS kindled rats compared to EH kindled rats (F_(3,165)_ = 10.82, ##p = 0.001). **D** An example of a labeled TRN cell. Data are expressed as Mean ± SEM.

**Figure 4:**
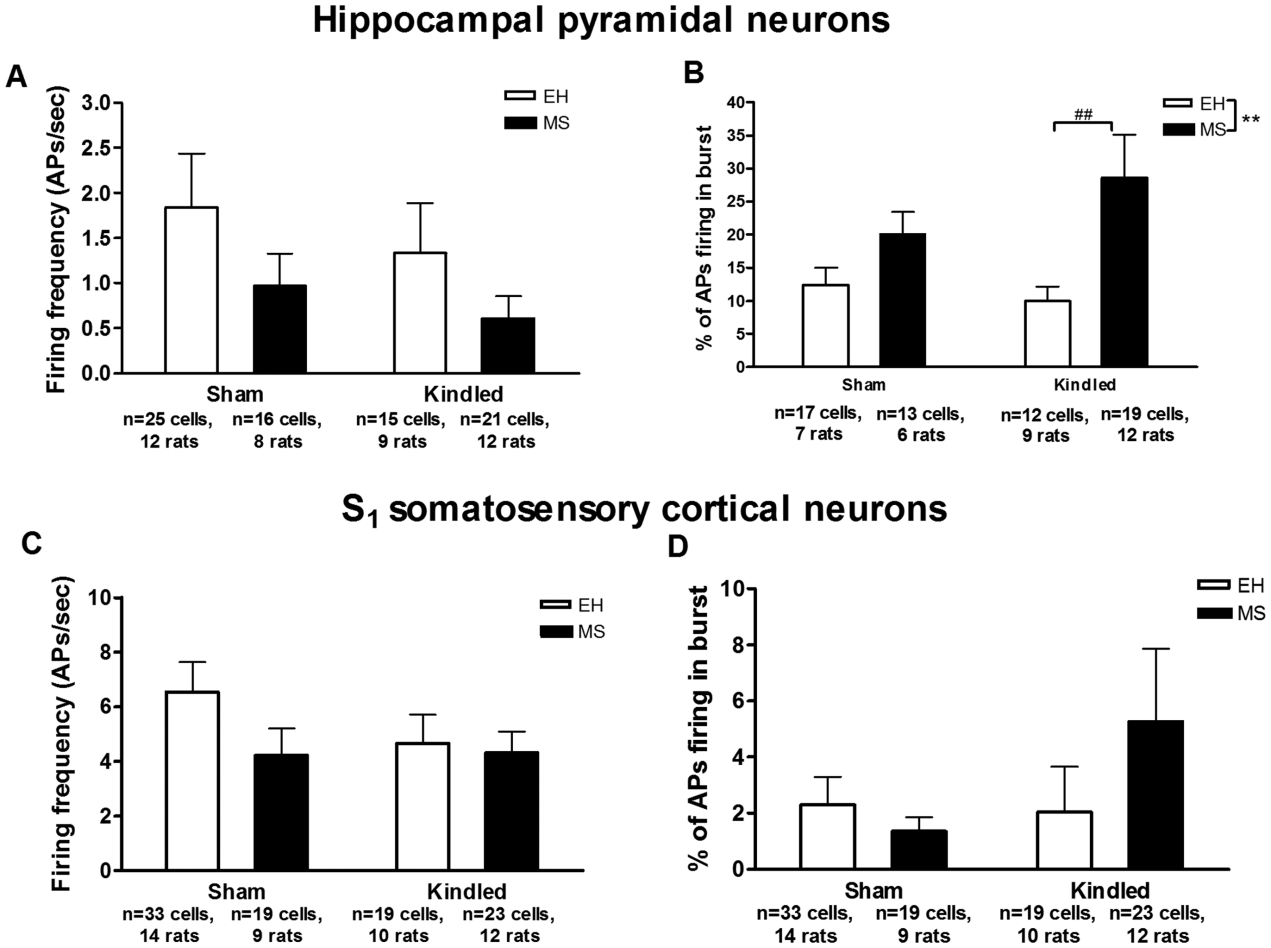
Effects of kindling and MS on interictal neuronal firing in hippocampus and somatosensory cortex. Neither MS nor kindling induced any effects on hippocampal pyramidal neuronal firing frequency (A), but an increase in the percentage of burst firing in this region was observed in MS rats (F_(3,74)_ = 10.54, **p = 0.001). Post hoc planned comparison showed that the percentage of action potentials firing in bursts were significantly more in MS kindled rats compared to EH kindled rats (F_(3,74)_ = 10.92, ##p = 0.001). In primary somatosensory cortical neurons, there was no significant effect of MS or kindling in **C** firing frequency or **D** percentage of action potentials firing in burst during interictal period. Data are expressed as Mean ± SEM.

MS as well as amygdala kindling led to a significant reductions in firing frequency of TRN neuronal cells (F_(3,168)_ = 5.41, p = 0.02 for MS and F_(3,168)_ = 8.93, p = 0.003 for kindling; Figure 3B). However, there was no significant effect of MS or kindling in the mean neuronal firing frequency of the hippocampal (F_(3,73)_ = 2.57, p = 0.11 for MS and F_(3,73)_ = 0.74, p = 0.39 for kindling, Figure 4A) or cortical neurons (F_(3,90)_ = 1.57, p = 0.21 for MS and F_(3,90)_ = 0.73, p = 0.39 for kindling, Figure 4C). When only the neurons that displayed burst firing (see below) were considered, the MS-induced reduction in firing frequency of hippocampal neurons approached statistical significance (p = 0.07), while no such effect was observed in the case of somatosensory cortical neurons (p = 0.18) (data not shown).

#### Burst firing

In TRN, there was no significant difference in the proportion of bursting cells between any of the treatment groups. Almost all the recorded TRN cells displayed bursts of high frequency action potentials (n = >2, interval of <6 ms) (Sham EH: 42/44 cells; Sham MS: 27/27; Kindled EH: 42/43 and Kindled MS: 57/57 cells had burst firing). However, in those cells that displayed burst firing, both kindling as well as MS were associated with a significant increase (F_(3,165)_ = 9.24, p = 0.002 for MS and F_(3,165)_ = 16.24, p<0.0001 for kindling) in percentage of action potentials which fired in bursts (Figure 3C). Post hoc planned comparison showed a significantly increased burst firing in kindled MS rats when compared to Kindled EH rats (p = 0.001). Kindling but not MS also altered characteristics of burst firing in the TRN (Table 1). Kindling was associated with a significant increase in mean number of action potentials firing in a burst (F_(3,165)_ = 8.18, p = 0.005), maximum number of action potentials in a burst (F_(3,165)_ = 6.75, p = 0.01) and intra-burst firing frequency (F_(3,165)_ = 4.10, p = 0.04). There was no effect of MS on all three characteristics of burst firing (Mean number of action potentials per burst: F_(3,165)_ = 0.00, p = 0.98, Maximum number of action potentials per burst: F_(3,165)_ = 0.41, p = 0.52 and Intra-burst frequency: F_(3,165)_ = 1.72, p = 0.19).

**Table 1:** Effects of MS and Kindling on characteristics of burst firing in the TRN.

	Sham		Kindled	
	EH (n = 42 cells, 14 rats)	MS (n = 27 cells, 9 rats)	EH (n = 43 cells, 13 rats)	MS (n = 57 cells, 12 rats)
**Mean number of APs in a burst**	3.5±0.2	3.4±0.2	4.0±0.2	4.0±0.2
**Maximum number of APs in a burst**	6.5±0.3	6.0±0.6	7.4±0.5	7.3±0.3
**Intraburst frequency**	285.7±9.7	302.6±16.0	310.9±10.0	323.4±10.0

Data are expressed as Mean ± SEM. Kindling led to a significant increase in mean number of action potentials (APs) per burst (F_(3,164)_ = 8.18, p = 0.005), Maximum number of APs per burst (F_(3,164)_ = 6.75, p = 0.01) and Intraburst frequency (F_(3,164)_ = 4.10, p = 0.04). MS did not affect any of the three characteristics of burst firing.

In the hippocampal region, Fisher's exact test showed a significant increase in proportion of the cells with burst firing in MS rats compared to EH rats (MS: 32/37 cells, EH 30 out of 40 cells; p = 0.005). This effect was not observed when comparing kindled versus sham rats (p = 0.09). In addition, considering only the cells that had burst firing, MS was also associated with a significant increase (F_(3,58)_ = 8.27, p = 0.005) in percentage of action potentials firing in bursts. Post hoc planned comparison showed that the percentage of action potentials firing in bursts were significantly greater in kindled MS rats compared to kindled EH rats (F_(3,58)_ = 10.92, p = 0.001; Figure 4B). Unlike MS, amygdala kindling did not have a significant effect on the percentage of action potentials firing in bursts (F_(3,58)_ = 0.47, p = 0.47). Both MS as well as kindling did not affect any of the three characteristics (mean number of action potentials in a burst, maximum number of action potential in a burst and intraburst frequency) of burst firing in hippocampus (Data not shown).

In the somatosensory cortex, no significant effect of MS or kindling on percentage of action potentials firing in bursts (F_(3,90)_ = 0.49, p = 0.48 for MS and F_(3,90)_ = 1.24, p = 0.26 for kindling; Figure 4D) or the characteristics of the burst firing were observed.

**Figure 5:**
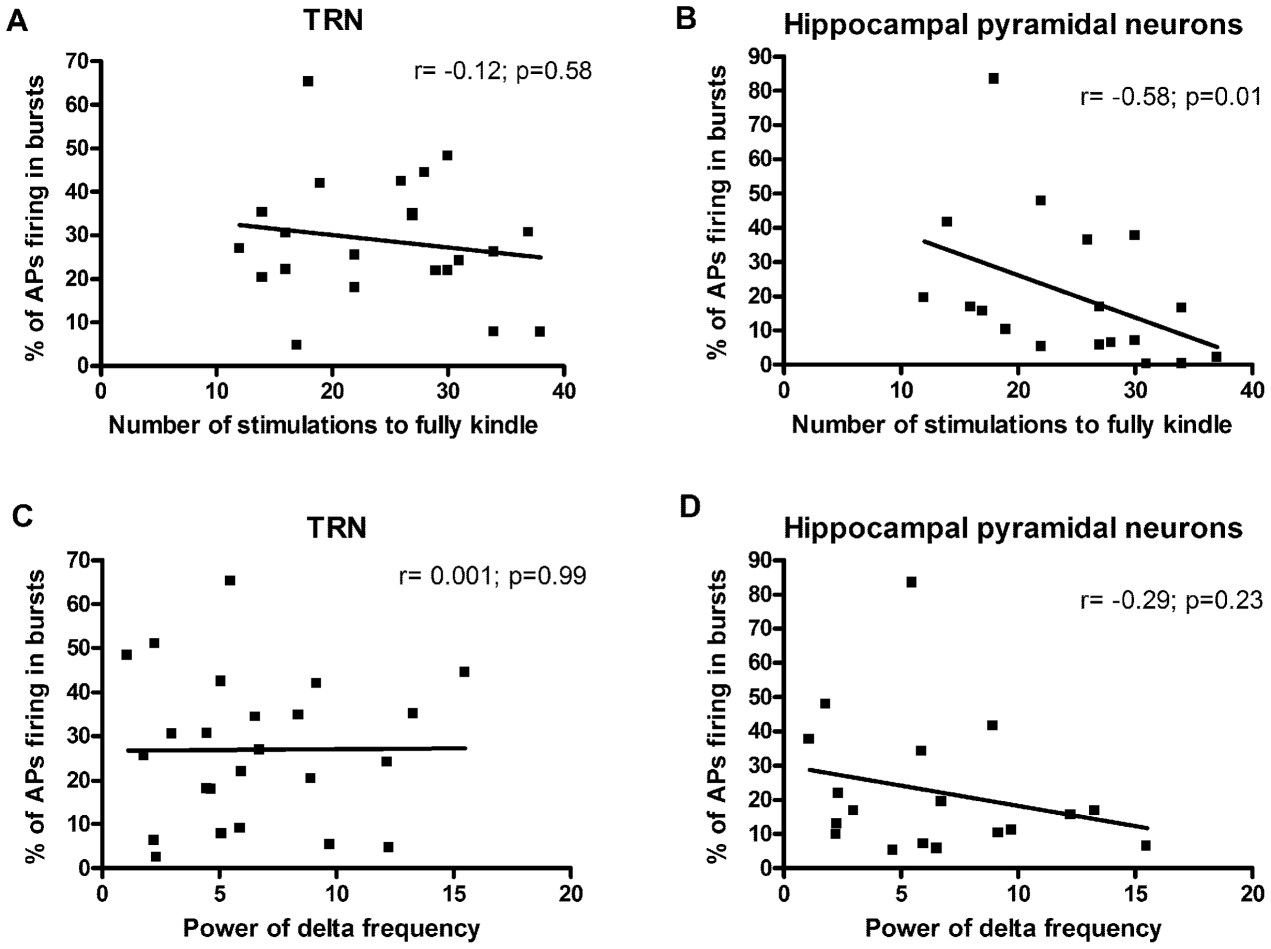
Correlation of burst firing with number of stimulations to reach fully kindled state and power of EEG delta frequency. The number of stimulations required to reach the fully kindled state was not correlated with the percentage of action potentials firing in bursts in the TRN (Spearman r value = −0.12, p = 0.58, A). While in the hippocampus, a significant negative correlation was observed between these two parameters (Spearman r value = −0.58, p = 0.01, B). No significant correlation was observed between the percentage of action potentials firing in bursts and power of EEG delta frequency for either the TRN (Spearman r value = 0.001, p = 0.99, C) or the hippocampus (Spearman r value = −0.29, p = 0.23, D).

The percentage of burst firing observed in the TRN and the hippocampal pyramidal neurons from all rats were correlated with the number of kindling stimulations delivered for each rat to reach a fully kindled state. This was done to assess whether the increase in the burst firing was related to the number of stimulations delivered to a rat to become fully kindled. When considering the TRN neurons, we did not find any significant correlation (Spearman r value = −0.12, p = 0.58 Figure 5A) between these two parameters. However, we observed a negative correlation (Spearman r = −0.58; p = 0.01) between these two parameters in the hippocampus, suggesting an inverse relationship between them (Figure 5B).

In addition, the percentage of burst firing in the TRN and hippocampal pyramidal neurons from all rats were also correlated with the power of EEG delta frequency observed in the frontal EEG. No significant correlation was found between these parameters for either the TRN or the hippocampus (Figure 5 C and D).

### Kindling in MS exposed rats enhances interictal rhythmicity in firing

**Figure 6:**
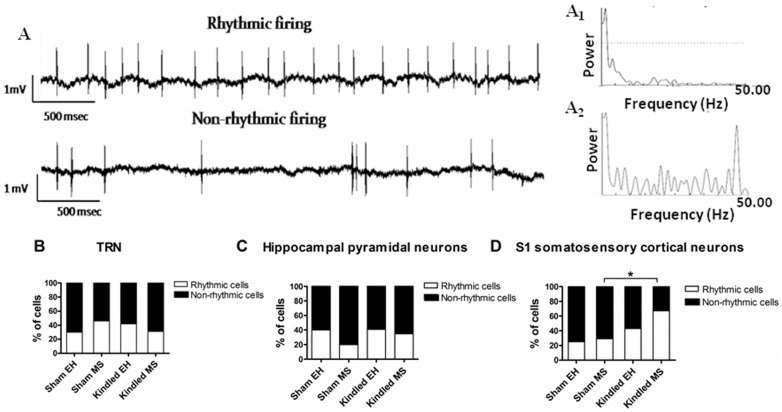
Effects of MS and kindling on percentage of cells recorded which fired in rhythmic pattern. Representative traces from a rhythmic and a non-rhythmic neuron are depicted in **A**. Examples of Lomb spectrogram are shown for **A_1_** rhythmic and **A_2_** non-rhythmic neurons. There was no effect of MS or kindling on rhythmicity of firing from **B** TRN or **C** hippocampal pyramidal neurons. **D** However, we did observe a significant overall increase in proportion of cortical cells firing in rhythm in kindled rats compared to non-kindled rats (p = 0.006). Post-hoc analysis revealed that the proportion of cortical cells rhythmically firing in kindled MS rats was significantly more compared to sham MS rats (p = 0.02).

The effect of early life stress and amygdala kindling on interictal rhythmicity of neuronal firing was investigated in hippocampal, TRN and somatosensory cortical neurons. We found a significant increase in proportion of the neurons that were firing rhythmically (Fisher's exact test; p = 0.006) in the S_1_ somatosensory cortex of kindled rats (25 rhythmic cells from 45 cells that were analysed) compared to sham rats (13 rhythmic cells from 48 cells). While considering only MS rats, the proportion of cells rhythmically firing in kindled MS rats was significantly more compared to the sham MS rats (p = 0.02). A similar effect was not observed while considering only EH rats (kindled EH versus Sham EH rats; p = 0.2; Figure 6D). In TRN or hippocampus, unlike cortical neurons, there was no significant effect (p>0.05) of MS or kindling on the proportion of neurons that were displaying rhythmic discharges (Figure 6B and C).

### Effects of MS and kindling on neuronal firing patterns during the progression of a stimulation-induced seizure

**Figure 7:**
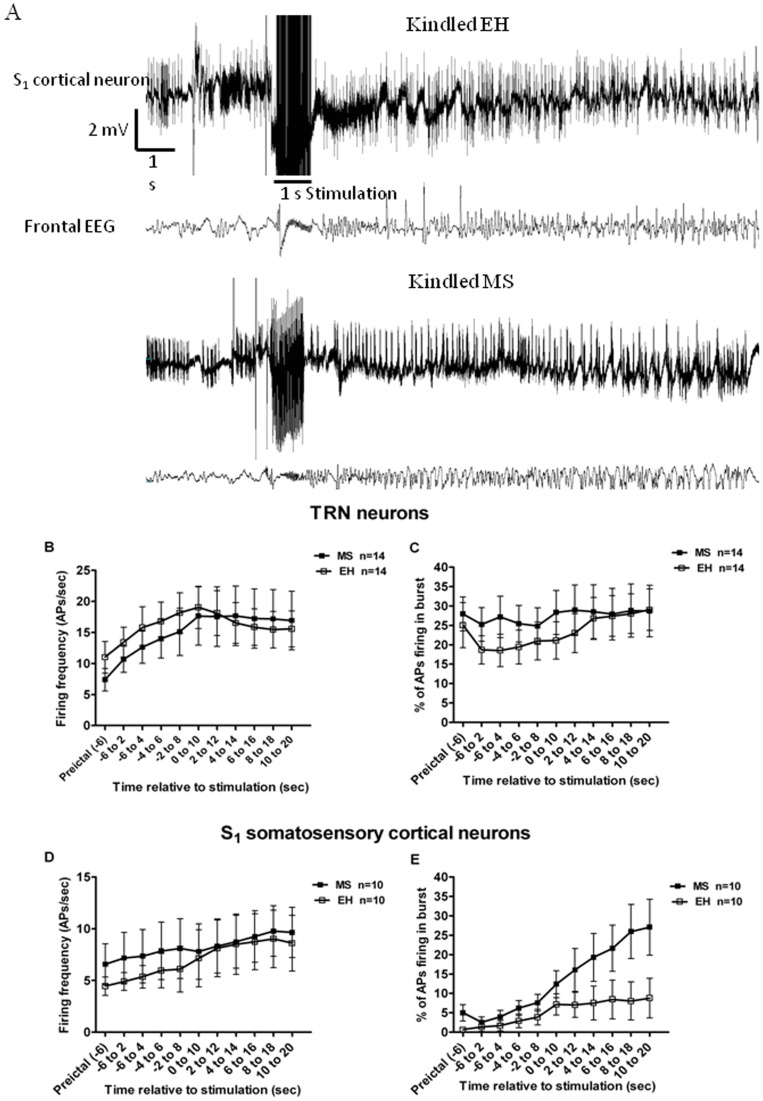
Analysis of neuronal firing patterns during progression of a stimulated (kindled) seizure. Representative recordings of somatosensory cortical neurons before and after electrical stimulation are shown in **A** (EEG is included as the lower trace). **B** In TRN, a significant increase in firing frequency (effect of time F_(1,14)_ = 7.17, p<0.001) with the progression of seizure was observed, but there was no effect of ELS on this parameter. **C** Percentage of action potentials (Aps) firing in bursts were not affected by ELS or progression of seizure (F_(1,14)_ = 0.07, p = 0.78). **D** In somatosensory cortex, there was a significant increase in neuronal firing frequency with the progression of seizures (effect of time F_(1,10)_ = 2.97, p = 0.001) but no significant effect of MS was observed (F_(1,10)_ = 0.16, p = 0.69). **E** There was a significant increase in percentage of action potentials firing in bursts with progression of seizures (effect of time; F_(1,10)_ = 8.92, p<0.000). This increase in the burst firing during the seizures was more pronounced in MS rats compared to EH rats (Interaction: MS × time; F_(1,10)_ = 2.38, p<0.011). Data are expressed as Mean ± SEM.

The effects of MS were investigated on neuronal firing patterns during the initiation and progression of an electrically evoked seizure. In TRN neurons, an increase in the firing frequency in both MS and EH rats was recorded with the progression of the seizure (F_(1,14)_ = 7.17, p<0.001; Figure 7B), but there was no significant effect of MS (F_(1,14)_ = 0.07, p = 0.78). The percentage of action potentials firing in bursts were not affected by either MS (F_(1,14)_ = 0.44, p = 0.51) or by the progression of seizure (F_(1,14)_ = 1.89, p = 0.055: Figure 7C).

In S_1_ somatosensory cortical neurons, mean neuronal firing frequency significantly increased with the progression of the seizure (effect of time F_(1,10)_ = 2.97, p = 0.001), but there was no significant difference in the firing frequency between MS and EH rats (F_(1,10)_ = 0.16, p = 0.69; Figure 7D). Similarly, there was a significant increase in the percentage of action potentials that fired in bursts with the progression of seizure (Time; F_(1,10)_ = 8.92, p<0.001). This increase in the burst firing during the progression of a seizure was significantly more in the MS rats than in EH rats (Interaction: stress × time; F_(1,10)_ = 2.38, p<0.01; Figure 7E). The hippocampal data are not presented due to unavailability of sufficient numbers of hippocampal recordings during an evoked seizure.

## Discussion

This study investigated the effect of early life stress induced by MS and amygdala kindling on *in vivo* neuronal firing patterns in brain structures relevant to the progression of limbic seizures induced by amygdala kindling. The primary findings were: (i) interictally, in the TRN, kindling and MS were associated with lower frequency of tonic firing and with increased burst firing; (ii) interictally, in hippocampal pyramidal neurons, MS, but not kindling, was associated with a greater percentage of action potentials firing in bursts; (iii) in somatosensory cortex, during a stimulated seizure, MS rats had a more pronounced increase in burst firing of neurons compared to EH rats.

Early life stress has enduring effects on brain electrophysiological functioning, neuronal plasticity, and neurochemistry in structures relevant to limbic epilepsy and its comorbid affective disturbance, notably the hippocampus [3], [4], [30]. The increased burst firing observed in this study in the hippocampus in MS exposed rats could strengthen synaptic neurotransmission [31], [32] by facilitating neurotransmitter release and signal transmission [33], [34], thereby increasing excitability of local circuits. Increased burst firing in hippocampus and midline thalamic regions has been implicated in the pathogenesis of MTLE [35], [36], [37]. In addition, in 5HT_1A_ receptor knockout mice, which display an anxiety-like phenotype, enhanced excitability in the hippocampal CA1 subregion has been reported [38], [39]. Furthermore, pharmacological inhibition of synaptic transmission in CA1 region of the hippocampus produces anxiolytic effects [40], [41]. Thus, the enhanced excitatory phenotype of hippocampal pyramidal cells observed in rats exposed to MS may also be relevant to their greater anxiety-like behaviour.

We found that kindled rats had a more bursting (epileptiform) interictal pattern of neuronal firing of TRN neurons compared to sham kindled rats. Several studies have suggested a role for thalamic structures in the development and progression of limbic seizures [17], [42], [43], [44]. Enhanced burst firing has been shown in neurons of the midline thalamic nucleus of spontaneously seizing rats following pilocarpine-induced status epilepticus (SE) [36], [42]. Moreover, the TRN has been directly implicated in the development of limbic seizures: simultaneous electrical stimulation of the TRN slows kindling progression along with the severity of hippocampal kindling seizures [18]; while focal injection of the muscarinic agonist, carbachol, in the TRN elicits limbic and generalised convulsive seizures [19], [20]. Hence, kindling-induced bursting pattern in TRN neurons could be part of the neuronal mechanisms underlying secondary generalization of limbic seizures. We observed a similar pattern of neuronal firing, i.e. slower but with enhanced burst firing, in TRN neurons of rats exposed to MS compared to EH. Burst firing in the TRN of kindled rats exposed to MS was significantly more than in kindled EH rats. Taken together, these findings suggest that MS has effects on neuronal firing properties of TRN that are qualitatively similar to, but not as marked as, those induced by amygdala kindling. If this cellular phenotype contributes to the progression of kindling epileptogenesis, this could be part of the mechanism for faster progression of kindling in rats exposed to MS.

A number of possible mechanisms may underlie the enhanced burst firing observed in kindled and MS exposed rats, including increases in persistent sodium currents [45], enhanced expression and function of T-type calcium channels [37], [46], [47], calcium-dependent small-conductance-type 2 (SK2) potassium channels [48] or increased levels of corticotropin releasing hormone (CRH) [49]. The properties of these neuronal bursts can vary according to the related mechanisms; for instance bursts can be of longer or shorter duration depending on the activation of particular T-type calcium channel isoforms [50]. In contrast to the effects of kindling in the current study, burst firing properties such as mean and maximum number of action potentials within a burst were not affected by MS. This may suggest a possible difference in mechanisms associated with the generation of burst firing induced by MS and by amygdala kindling.

It is also critical to understand how MS mediates its effects in thalamic structures. The TRN is known to have connections with the limbic structures via midline thalamic regions such as the nucleus reuniens [51], [52]. Effects of early life stress have been widely described in limbic structures and in several midline thalamic structures including paraventricular nucleus of thalamus and anterodorsal thalamic nuclei [53], [54], [55]. However, direct studies reporting effects of stress in lateral thalamic structures are scarce. One study reported increased mRNA and protein expression of CRH, an important mediator of stress with predominantly excitatory effects in the brain [56], [57], and its receptor CRHR_1_ in thalamus of rats exposed to MS [58]. In addition, a study conducted in rhesus monkeys showed an increased thalamic glucose metabolism that corresponds to increased neuronal activity, in maternally separated monkeys compared to the non-separated controls [59]. Moreover, TRN of rats exposed to chronic stress in adulthood displayed increased expression of activity-regulated cytoskeleton-associated protein (Arc), an immediate early gene that represents enhanced neuronal activity [60]. Another study (unpublished data discussed in a review [61]) showed reduced α2 subunit protein expression of GABA receptors in lateral-dorsal thalamic regions and somatosensory cortex of rats exposed to early life stress. Alterations in α subunits of GABA receptors could alter its receptor pharmacology and function [62], [63]. Some of these molecular and metabolic alterations may be related to the effects of early life stress on electrophysiology in the lateral thalamic structures we observed.

In addition to the effects on single cell neuronal firing, we also investigated the effects of MS and kindling on brain neuronal network function by examining the EEG activity recorded over the cortex. Amygdala kindling was associated with a significant increase in the power of the delta frequency band both in rats under neuroleptic analgesia and in freely moving rats, consistent with previous studies showing increased delta activity in the amygdala of kindled rats [64], [65]. Nazer and Dickson [66] showed that slow oscillations in the EEG promote epileptiform discharges in the hippocampus. Increased delta activity has also been reported to be related to the increased neuronal burst firing [67], although in this study we found no correlation between these two measures for either the hippocampus or the TRN (Figure 5C&D). The absence of correlation in these measures likely reflects the absence of functional relation between the recording sites in frontal cortex and the hippocampus as well as the TRN. In freely moving rats exposed to MS, there was a significant decrease in the delta frequency EEG activity. The implications of this finding are unknown but, interestingly, it is in line with decreased EEG power over wide frequency bands associated with early stress in humans [68].

We also investigated the effect of MS on neuronal firing with the onset and progression of a kindled seizure in the TRN and somatosensory cortex. With the onset of a seizure there was an increase in firing frequency as well as burst firing of somatosensory cortical neurons, whereas in TRN there was an increase in firing frequency only. A similar pattern has been reported previously *in vivo* in the amygdala [69], piriform and perirhinal cortices [70] and substantia nigra pars reticulata [71], and also in hippocampus with chemoconvulsant induced seizures [72], [73]. Our results are consistent with *in vitro* findings from epileptic rats of increased burst firing in somatosensory cortex in brain slices [37]. However, ours is the first study to investigate neuronal firing patterns in the somatosensory cortex during an amygdala kindling-induced seizure. Notably, we found that the increase in burst firing with the progression of a limbic kindled seizure was significantly greater in rats previously exposed to MS. Such enhanced propensity to burst firing of cortical neurons may contribute to the increased propensity of animals exposed to MS to develop generalized convulsive seizures.

Overall, the results of this study demonstrate that exposure to early life stress in the form of MS induces enduring alterations in the firing patterns of neurons in the hippocampus, TRN and somatosensory cortex that may help explain the increased vulnerability to progression of limbic epileptogenesis brought by MS. These changes were more marked following kindling, and neurons in the somatosensory cortex were more vulnerable to being recruited into a burst firing pattern during a kindled seizure in rats that had experienced MS. Further studies aiming to understand the molecular mechanisms underlying the generation of burst firing observed in this study could provide support for a causal relationship between electrophysiological changes in epilepsy circuits and epilepsy progression, and open up potential therapeutic options involving pharmacological inhibition of these pathways.
